# Self-Driven Drug Release Dual-Layer Dressing Composed of Electrospun Membrane and Hydrogel with Dynamic Structure Based on Piezoelectricity Transformation and Electrical Responsiveness

**DOI:** 10.3390/polym18172175

**Published:** 2026-09-06

**Authors:** Qiaoling Wu, Yanping Zhao, Wenqian Zhu, Fengzhu Lv, Gao Si

**Affiliations:** 1 Engineering Research Center of Ministry of Education for Geological Carbon Storage and Low Carbon Utilization of Resources, School of Materials Science and Technology, China University of Geosciences, Beijing 100083, China; 18985803968@163.com (Q.W.); ypzhao0423@163.com (Y.Z.); wenqianzhu@email.cugb.edu.cn (W.Z.); 2 Department of Orthopaedics and Engineering Research Center of Bone and Joint Precision Medicine, Peking University Third Hospital, Beijing 100083, China

**Keywords:** self-powered dual-layered dressing, piezoelectricity, self-healing, dynamic structure change

## Abstract

This study reports the development of an intelligent dual-layer wound dressing engineered to achieve active wound repair. The self-standing dressing integrates a polyvinylidene fluoride (PVDF) piezoelectric electrospun membrane with a multifunctional hydrogel based on interfacial fusion. The PVDF layer converted mechanical energy derived from human body movement into electrical stimulation, which dramatically motivated the controlled release of diclofenac sodium (DFs) encapsulated in the hydrogel. The relatively small diameter fibers and network caused strong connection with the hydrogel and smooth transfer of electricity. When a positive potential of 1.5 V was applied, up to a 55 μg mL^−1^ cumulative amount of DFs was released, about five times higher than that only based on drug diffusion. This enhanced release rate resulted from the cooperation of accelerated drug migration under electrically driven and dynamic changes of the gel’s microstructure, which was the result of reversible behavior of borate bonds within the hydrogel. Consequently, the hydrogel exhibited self-healing properties and a tensile strain of up to 600%, much higher than that of conventional hydrogels. Under mechanical motivation, the dressing exhibited enhanced DFs release ability, confirming the piezoelectric transition and controlled drug release abilities. Therefore, the present work offers an innovative solution for the intelligent management of chronic wounds.

## 1. Introduction

Drug delivery can effectively enhance the efficacy of drug therapy and reduce systemic side effects [1,2]. Conventional hydrogels, with their excellent biocompatibility and three-dimensional porous network structure, enable local drug retention and sustained release. However, such materials lack environmental responsiveness, and their mechanical properties and controllability of drug release are limited, making them unable to meet the demands of precision medicine [3]. For example, physical encapsulation of drugs in conventional hydrogels not only limits the hydrogel’s capacity but also facilitates burst release effect at the original range, followed by a sharp decline in release rates as drug concentrations decrease, resulting in a narrow therapeutic concentration window. Moreover, their release kinetics are highly dependent on passive diffusion driven by concentration gradients [4]. This makes it difficult to meet the treatment demands of chronic wounds requiring sustained, steady drug delivery over extended periods.

Driven by the clinical pursuit of precision and personalized medicine, stimulus-responsive smart drug-delivery systems for wound management have witnessed rapid advances [5,6]. Various stimuli-responsive wound dressings have been well-developed, including light-responsive drug-delivery platforms, thermoresponsive hydrogels and magnetically responsive systems [7,8,9]. Light-responsive systems require external light irradiation and suffer from limited tissue penetration depth, accompanied by potential overheating risks. Magnetically responsive platforms rely on external alternating magnetic field equipment for activation. Thermoresponsive hydrogels are triggered by temperature variation instead of endogenous biomechanical motion.

Among the various stimulation-responsive modes, electrical stimulation has demonstrated excellent potential for application in smart on-demand drug delivery systems. This approach enables the remote and precise control of drug release doses and duration, thereby effectively enhancing therapeutic efficacy; at the same time, electrical signals possess excellent programmability, allowing for the dynamic regulation of drug release behavior in accordance with actual clinical needs. In the electrical stimulation system, electro-responsive drug delivery greatly relies on functionalized conducting polymers for accurate in situ drug release. For example, Lin et al. [10]. reported a biocompatible hydrogel electronic patch for diabetic wound therapy. The PEDOT: CHC/silk conductive hydrogel, constructed via amphiphilic chitosan doping and silk-fibroin cross-linking, possesses a hierarchical structure for high loading efficiency (>90%) of hydrophobic ibuprofen. Programmable drug release can be realized by tuning electrical stimulation waveforms, intensity, frequency and time.

Conductive hydrogels are promising in forefront areas because of the joint merits of softness, tunable drug-carrying capacity and conductivity [11,12]. Thus, conductive hydrogels are considered ideal carrier platforms for achieving highly efficient electrically stimulated drug delivery. Li et al. [13] proposed a flexible, non-cytotoxic drug-loaded electret based on carnauba wax (nCW) nanoparticles. The electret loaded with curcumin exhibited ultrasonic and electrical stimulation responsiveness, enabling precise controlled drug release. This research offers novel insights into the construction and testing of bio-electret materials, demonstrating broad application potential in the field of on-demand precision drug delivery. Lv et al. [14] proposed a polyacrylamide nanocomposite conductive hydrogel based on hydrogen bonds and borate ester bonds, exhibiting high ionic conductivity, excellent biocompatibility, and antibacterial properties. Compared to existing materials, this hydrogel achieved rapid electrical response and enhanced drug release. This non-invasive system enables stable, long-term, and precise drug delivery, offering a novel therapeutic strategy for chronic skin diseases. Moreover, Huang et al. [15] developed a single-layer conductive wound dressing (SR-Gel/ZL) by incorporating levofloxacin-loaded ZIF-8 nanoparticles (ZIF-8@Levo) which can be used not only to treat bacterial infections but also simultaneously sense and distinguish between temperature, blood-related signals and pressure. The conductive hydrogels maintain tight adhesion while efficiently converting external electrical signals into energy that drives drug release through their embedded conductive networks, enabling active and intelligent management of drug release kinetics [16,17]. However, existing electrical stimulation delivery systems typically rely on bulky external power sources and complex wiring connections [18], which severely restricts patients’ daily activities, reduces wear comfort, and limits the feasibility of long-term use.

A piezoelectric nanogenerator (PENG) could convert the body’s subtle movements, such as a light pat of the hands or slight limb movement, into electrical energy, enabling wearable sensors to operate without the need for an external power source [19,20,21,22]. Furthermore, mechanical motion enables flexible regulation of output voltage and power for diverse application scenarios. Fu et al. [23] proposed an innovative wound treatment system based on the concept of self-powering. This self-powered intelligent drug delivery system consists of two functional modules. One is a drug-loaded hydrogel, and the other is a power supply unit based on a wearable DC-pulsed PENG made of P(VDF-TrFE), which converts the patient’s movement into electrical energy. This approach avoids the potential risks associated with continuous drug delivery, thereby safely and efficiently accelerating wound healing. Different from the single electric field-responsive drug release of conventional PENG systems, our proposed hydrogel innovatively incorporates dynamic boronate ester covalent bonds to endow the material with reversible and tunable microscopic network structures. The dynamic characteristics of boronate ester bonds effectively optimize internal drug diffusion pathways, enable excellent mechanical adaptability to dynamic wound microenvironment changes, and confer superior microenvironment responsiveness.

In the field of flexible soft PENGs, PVDF and its copolymers have become core functional materials owing to their excellent piezoelectric response, good flexibility and processability [24,25,26]. In addition, polymers such as polylactic acid, nylon-11 and polyacrylonitrile are also widely used in wearable sensors, smart wound dressings and implantable devices due to their piezoelectric activity, biocompatibility or biodegradability, providing a rich selection of materials for the development of self-powered systems. Sun et al. [27] designed a self-powered single-layer film for accelerating the healing of infected wounds using PVDF as the matrix and carbonylated carbon nanotubes (c-MWCNTs-VAN) as additives, utilizing a ‘Lock-On/OFF’ electric field (EF) to drive drug release in combination. This work offered a new strategy for the precise and effective treatment of infected wounds. Subsequently, Sun et al. [28] developed a self-powered, thermoelectric-enhanced photothermal wound dressing comprising a PVDF piezoelectric layer (produced via wet spinning) on top and a PAM-based hydrogel layer underneath. By leveraging the excellent piezoelectric properties of PVDF, this design overcomes the impedance limitations at the interface between the two layers. It combines photothermal, thermoelectric and piezoelectric effects to synergistically regulate and reshape the endogenous electric field within the wound, thereby accelerating tissue repair. The above demonstrates that PVDF can provide an electric field output for dressings, while hydrogels conform to the wound site and exhibit good biocompatibility; the combination of the two is driving the clinical application of smart wound dressings.

To further decrease the interface impedance, PVDF electrospun membrane was combined with structure-adjustable and conductive hydrogel to produce a Janus structure dressing with self-driven and controlled drug release behaviors. The proposed bilayer PVDF piezoelectric membrane–hydrogel platform integrates the high drug-loading capacity of hydrogels and the self-powered feature of piezoelectric membranes. Meanwhile, the engineered interfacial fusion builds a stable bilayer structure, avoiding interfacial delamination and signal instability under mechanical deformation while still containing a porous structure, affording strong mechanical strength and breathability for the dressing. Moreover, structure-adjustable hydrogel was fabricated by constructing reversible boronated bonds between bio-friendly and charge-containing SA and PVA with drugs embedded inside. Dynamic structural change of the hydrogel and the accelerated migration of drugs by electricity resulted in controllable and accelerated drug release. Under a 1.5 V positive potential, a high, up to 55 μg mL^−1^, cumulative release amount of DFs was obtained with the hydrogel, about five times higher than that only based on drug diffusion. Under mechanical pressure, the dressing also indicated prominent drug release ability compared to the control sample. Thus, this novel dressing is capable of self-sustaining energy generation, enables precise and controlled drug release, and combines long-lasting antibacterial and anti-inflammatory effects with wound healing in a single integrated solution.

## 2. Materials and Methods

### 2.1. Materials

Sodium alginate (SA), Polyvinyl alcohol (PVA, Mn = 80,000–1,000,000), borax (sodium tetraborate pentahydrate), tannic acid (TA), and *N*, *N*-dimethylformamide (DMF, AR) were obtained from the Shanghai McLean Reagents Co., Ltd. (Shanghai, China). Polyvinylidene fluoride (PVDF, MW = 600,000), Ag nanoparticles (AgNPs, 50 nm), and all other chemicals were obtained commercially from Beijing Chemical Reagent Factory (Beijing, China) and used as received.

### 2.2. Preparation of PVDF Electrospun Nanofibers

PVDF (0.6 g) was dispersed in 5 mL of DMF and stirred at 40 °C for at least 6 h until a uniform milky white solution was obtained. Electrospinning was carried out with a nozzle-to-collector distance of 18 cm, a voltage of 20 kV and an injection rate of 1 mL/h. The resulting membrane was dried in an oven at 40 °C for 6 h to yield a porous PVDF piezoelectric membrane.

### 2.3. Preparation of PBST-xAg-DFs Hydrogels

The PBST-xAg-DFs composite hydrogel [29] was synthesized using the following procedure. PVA in an amount of 0.85 g was dissolved in 10.6 mL of deionized water at 90 °C to give a PVA solution. Then, 0.3 g of SA, 2.4 mL of TA aqueous solution (40 mg/mL) and 1 mL (0.01 g/mL) of DF were added into the PVA solution in turn as the previous component dissolved completely. Then, an appropriate amount of AgNPs was introduced, followed by continuous ultrasonication for 30 min. Finally, the required dose of borax was added after 3 min to the above system to obtain the PBST-xAg-DFs drug-loaded conductive hydrogel, where x indicates the weight percent of AgNPs in the hydrogel.

### 2.4. Preparation of Self-Driven Wound Dressing

In practice, the electrospun membrane was placed at the bottom of the mold, with the precursor of hydrogel casting over it. Then cross-linking agent was sprayed on it. After curing for 1 h, a tightly connected bilayer structure was formed.

### 2.5. Drug Release

Drug release from the hydrogel or dressing was evaluated using two methods. One employed an electrochemical workstation in a three-electrode system, and the other utilized a Franz diffusion cell in an ultrasonic cleaner.

#### 2.5.1. Drug Release Under Electrical Stimulation

To investigate the drug release behavior of the samples under applied electrical stimulation, this experiment employed a three-electrode system using an electrochemical workstation. Drug-loaded hydrogels and wound dressings were used as the working electrodes, a platinum foil as the counter electrode, and an Ag/AgCl electrode as the reference electrode. PBS buffer solution was selected as the electrolyte to simulate the human body fluid environment. Potentials of 0 V, 0.5 V, 1 V and 1.5 V were applied to the DFs-loaded hydrogel for 260 min. The cumulative amount of drugs released into the PBS solution was tested based on UV–Vis measurement and calculated based on the standard curve. Once the power is switched on, 3 mL of supernatant is removed from the beaker every 10 min, and the absorbance of the DFs is measured at a wavelength of 276 nm using UV–Vis spectrophotometry. Once the measurement is complete, the supernatant is returned to the original beaker to ensure that the volume of the release system remains constant.

#### 2.5.2. Piezoelectricity-Driven Drug Release

The release of the dressing through the simulated skin under a piezoelectricity-driven platform was carried out in a Franz diffusion cell in an ultrasonic cleaner [30]. A square dressing sample with a side length of 2 cm was taken and applied tightly to the surface of a cellulose film serving as a skin simulant. The cellulose film was secured to the upper end of the receptor chamber of the diffusion cell, with the effective permeation area matching the cross-sectional area of the dressing exactly. The receptor chamber was filled with deionized PBS solution to ensure that the cellulose film was fully saturated and adhered to the receiving solution, thereby providing a stable environment for the sustained trans-membrane permeation of the drug. In vitro drug permeation experiments were conducted under ultrasonic piezoelectric stimulation. Every 10 min, 3 mL of supernatant was aspirated from the receiving system, and the absorbance was measured at a characteristic wavelength of 276 nm using a UV–Vis spectrophotometer. Upon completion of the test, the aspirated supernatant was returned to the original system to maintain a constant total volume of the receiving solution, thereby minimizing the impact of volume fluctuations on the test results.

### 2.6. Antibacterial Assay

The biocompatibility of the hydrogels was tested based on the improved Kirby–Bauer methods. Nutrient agar (NA) was employed as the culture medium. Different hydrogel samples were co-cultured with *Escherichia coli* and *Staphylococcus aureus* in NA medium. After incubation at 37 °C for 12 h, the growth of the inhibition zones was observed.

### 2.7. Characterization Methods

Cross-sectional morphology of the hydrogel and PVDF nanofiber was obtained using a field emission scanning electron microscope (SEM, HITACHI-4800 (Tokyo, Japan)). For observation, the hydrogel was freeze-dried and coated with gold sputtering for 1000 s. The chemical compositions of the hydrogel were analyzed using Fourier transform infrared spectroscopy (FT-IR, Hitachi F-4700 Fourier (Tokyo, Japan)) within the range of 4000–500 cm^−1^. For PVDF, the range was 600−2000 cm^−1^. The total fraction of polar crystalline α and β phases for PVDF was calculated based on the FT-IR absorbance at 837 cm^−1^ and 763 cm^−1^ using the following equation [31,32,33]:

$$F\left(\beta \right)=\frac{A_{\beta }}{1.3A_{\alpha }+A_{\beta }}$$

where Aα and Aβ correspond to the absorbance values at 840 cm^−1^ (β phase) and 763 cm^−1^ (α phase). The constant 1.3 is an empirical value from the literature representing the ratio of absorption coefficients for PVDF β- and α-crystals.

XRD patterns were measured by an X-ray diffractometer (D/Max-2200, Rigaku, Japan) from 10° to 80° to detect the presence of the β phase in the nanofiber. X-ray photoelectron spectroscopy measurements (XPS, ESCALAB 250XI, Thermo Scientific (Waltham, MA, USA)) were applied to analyze the surface elemental composition and chemical states of B, C, and O. Tensile strengths of the samples were measured using a universal testing machine (ETM-304C) equipped with a 200 N load cell. Specimens were prepared as rectangular gauze sections (40 mm × 40 mm × 3 mm) and subjected to tensile testing at a loading rate of 100 mm min^−1^. The ionic conductivity of the hydrogel (2 × 2 × 0.3 cm) was measured via AC impedance spectroscopy (Electrochemical Workstation, CHI760E (Shanghai, China)) within a frequency range of 100 kHz to 1 Hz. Conductivity was calculated according to the following equation:

$$\sigma =\frac{1}{R}\frac{L}{A}$$

σ is the electrical conductivity in S·m^−1^, L denotes the thickness of the hydrogel, and A represents its effective cross-sectional area. R is determined by the intercept of the AC impedance curve at high frequency on the horizontal axis.

## 3. Results and Discussion

**Figure 1:**
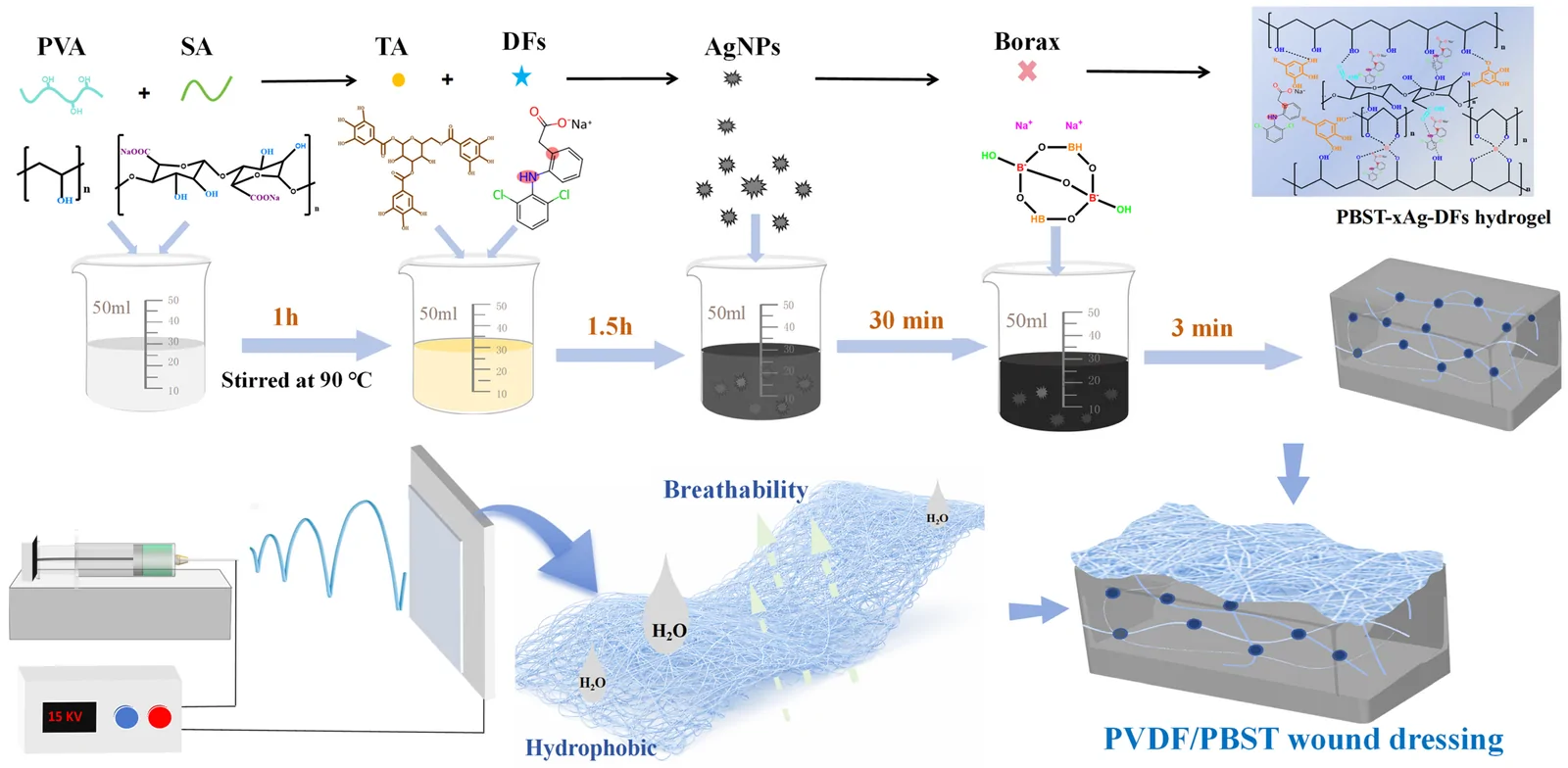
Schematic illustration of the preparation of self-powered wound dressing.

In the present work, conductive hydrogels were prepared utilizing biocompatible PVA and SA as the main raw materials and borax as the cross-linking agent through esterification cross-linking reaction. In the preparation process, DFs [26,34] with pain relief and anti-inflammatory effects was encapsulated in the hydrogels. Then, a piezoelectric electrospun PVDF membrane was coated on the surface of the hydrogels to form piezoelectric-driven patches just as shown in Figure 1. Thus, a self-driven Janus structure dual-layer dressing for chronic wound repair and on-demand drug delivery was successfully fabricated. Subsequently, the properties of the hydrogel and the dressing as well as the mechanism of drug release driven by electricity and mechanical pressing were systematically investigated.

### 3.1. Preparation and Properties of the Hydrogels

#### 3.1.1. Structure and Morphology of the Hydrogels

**Figure 2:**
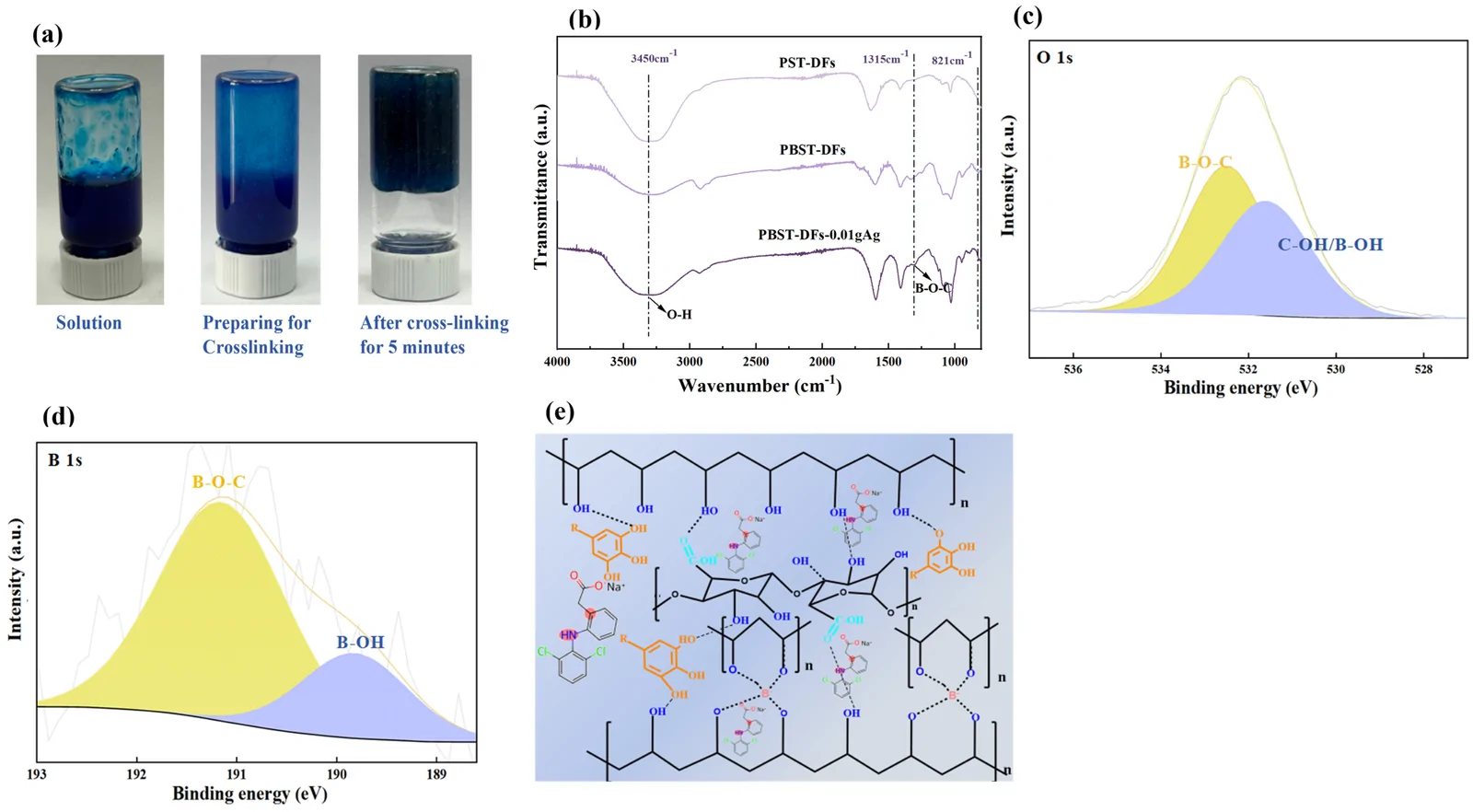
(**a**) Photos of PBST-0.01Ag-DFs hydrogel before and after cross-linking; (**b**) FT-IR, (**c**,**d**) XPS spectra and (**e**) possible interaction of different components in PBST-0.01Ag-DFs.

Figure 2a shows the successful formation of PBST-0.01Ag-DFs hydrogel, which had been colored by methylene blue for clear observation. After introduction of borax for 5 min, the gel became self-standing, and the shape of the gel could be maintained completely. The successful formation of the gel was also verified by FTIR, as shown in Figure 2b. The wide and strong bands at ~3450 cm^−1^ in all spectra were attributed to the O-H stretching belonging to PVA and SA. Compared with the spectrum of uncross-linked PBST-DFs, one characteristic peak at 1315 cm^−1^ was discovered in the spectra of PBST-DFs and PBST-0.01Ag-DFs. This characteristic absorption peak was attributed to the asymmetric stretching mode of the B-O-C bond, while the weak absorption peak observed at 821 cm^−1^ originated from the stretching vibration of the B-O bond. This confirmed the successful formation of a borate ester cross-linked structure within the system [29]. In summary, the three-dimensional network structure of the PBST hydrogel was constructed through the synergistic action of borate ester covalent bonds and hydrogen bonds. The XPS spectrum shown in Figure 2c,d further confirmed the chemical bonding characteristics of the hydrogel. The B 1s spectrum exhibited two characteristic peaks at 191.7 eV and 189.5 eV [35,36]. The former was attributed to the B-O-C, indicating that the hydroxyl groups of PVA formed a stable covalent cross-linked network with the boron-containing units, while the latter corresponded to unreacted B-OH, which provided active sites for hydrogen bonding. In the O 1s spectrum, the signal at 533 eV corresponded to the bridging oxygen of the boronate bond, corroborating the B 1 s results [37]. Just as illustrated in Figure 2e, the abundant catechol groups in TA, the phenolic hydroxyl groups in SA, and the hydroxyl groups (–OH) in PVA could form extensive covalent interactions with the cross-linking agent, simultaneously. Thus, boronate ester bonds formed between borax and PVA/SA, jointly constructing a three-dimensional network in which the drugs were uniformly dispersed.

**Figure 3:**
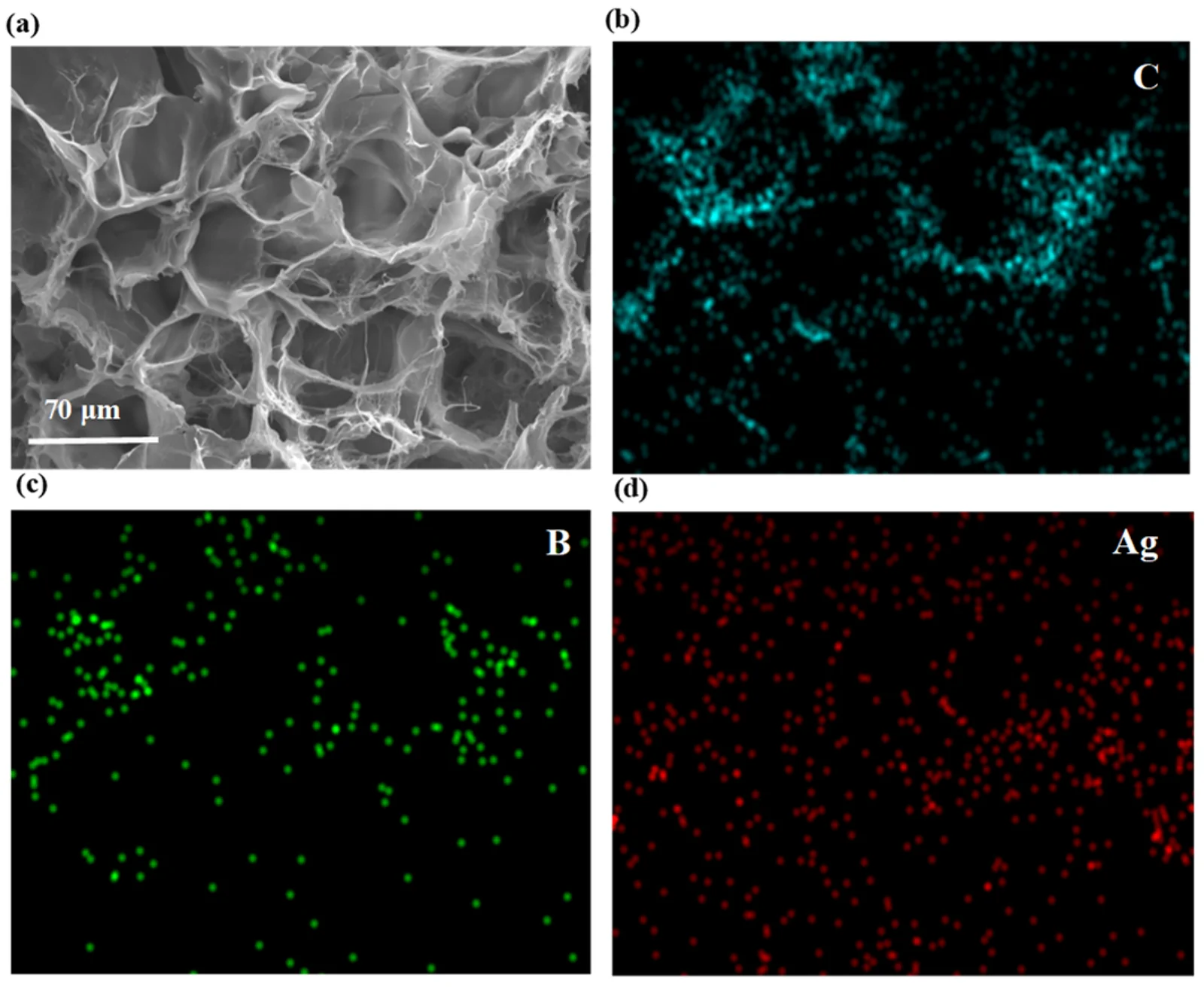
(**a**) SEM and elemental mapping of the hydrogel (**b**) C; (**c**) B; (**d**) Ag.

The cross-sectional morphology of PBST-0.01Ag-DFs was characterized by SEM and is shown in Figure 3a. PBST-0.01Ag-DFs hydrogel showed typical 3D network microstructure and irregularly distributed large pores, providing a theoretical basis for subsequent drug release. The elemental mapping results shown in Figure 3b–d confirmed that C, O, B and Ag were uniformly distributed within the hydrogel. The signal shapes of these elements were similar to that of the wall of hydrogel shown in SEM. No obvious large aggregation of Ag particles was observed. The material exhibited excellent compositional homogeneity.

**Figure 4:**
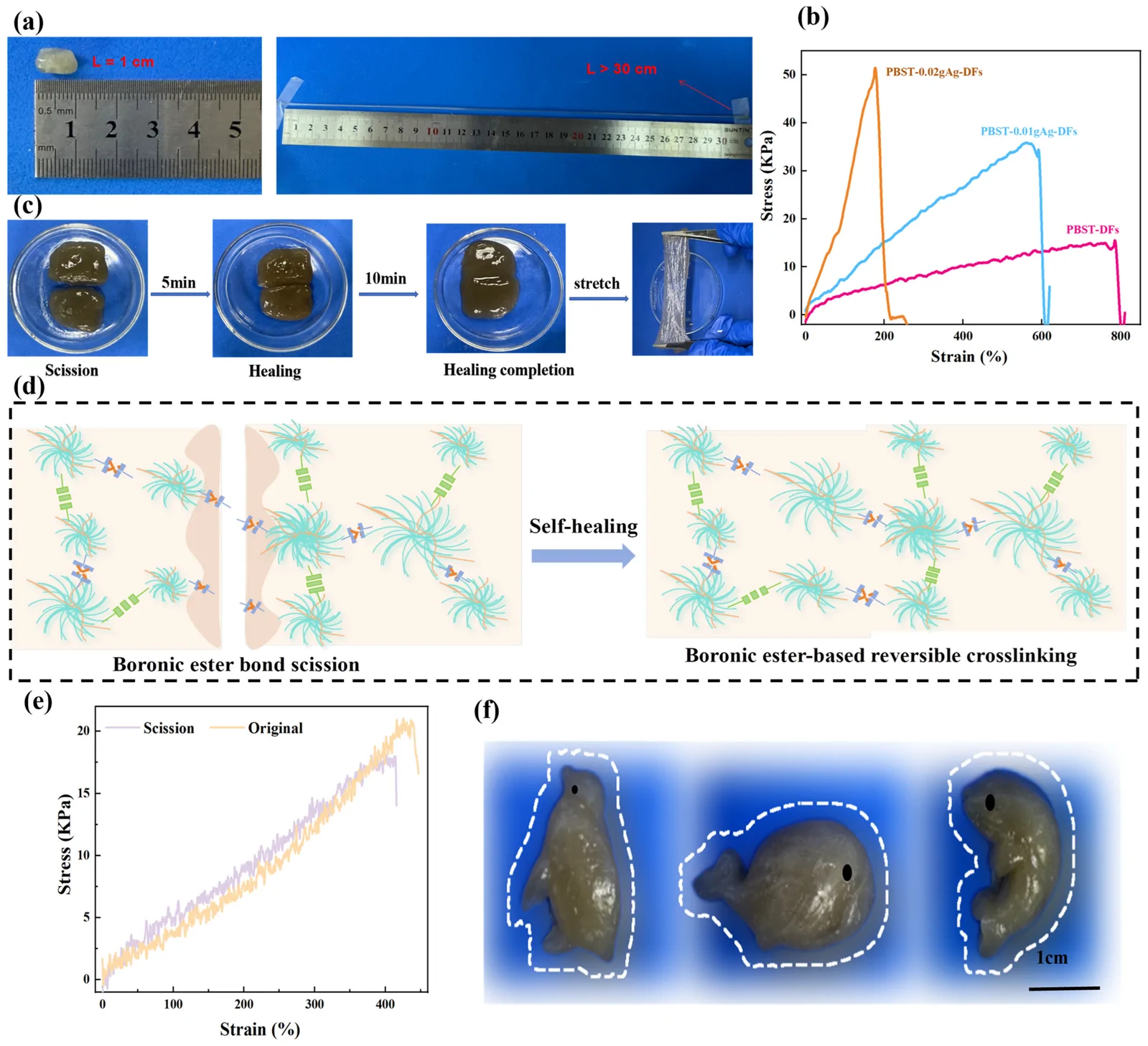
(**a**) Stretchability of the PBST-0.01Ag-DFs hydrogel; (**b**) Stress–strain curves of hydrogels with different AgNP content; (**c**) Time-lapse process of the self-healing of cracked PBST-0.01Ag-DFs; (**d**) Schematic diagram of PBST hydrogel self-healing; (**e**) Tensile stress curve of the incision-healing hydrogel compared with its original state; (**f**) Plasticity of the PBST hydrogel.

The tensile strength of PBST-0.01Ag-DFs was studied, as shown in Figure 4a. A 1 cm hydrogel rod could be stretched to more than 30 cm without break, and a more than 2900% stretching rate was obtained, observably demonstrating ultra-high tensile extensibility. The tensile strength was also measured by a universal testing machine, and the results are illustrated in Figure 4b. PBST hydrogel showed the highest tensile extensibility. With the introduction of AgNPs, the tensile strength was improved, while fracture strain was decreased. To obtain materials with both of the above properties, PBST-0.01Ag-DF was selected and studied further. This hydrogel exhibited a tensile strain of up to 600% and a modulus of approximately 30 kPa. The uniform network of the hydrogel enabled stable load-bearing under large deformations, offering greater durability and reliability.

To compensate mechanical damage, self-repairing properties constituted an attractive characteristic of the hydrogels. As shown in Figure 4c, PBST-0.01Ag-DFs hydrogel was cut into two segments and then set to contact. The two separated segments could self-heal into a complete one in about 5 min in an environment free from any external interference. Simultaneously, a comparison of the tensile properties between the self-healed hydrogel and the original hydrogel (40 mm × 20 mm × 2 mm) revealed a slight reduction in tensile strength, as observed in Figure 3d. But 95% of its tensile strength still could be recovered. The morphology of the surface vertical to the self-formed interface is shown in Figure S1. Uniform porous structure was observed. No obvious damaged pore walls were shown, indicating the self-adjustability of the hydrogel.

Figure 4d shows a schematic diagram illustrating the self-healing, which was due to the formation of borate ester bonds between the groups on the section surface of hydrogel. This autonomous self-healing ability also contributed to the hydrogel’s high stretchability [35]. This highlighted the PBST-xAg-DFs hydrogel’s stability and durability for practical applications. Owing to the intrinsic dynamic reversible chemical bonds, the as-prepared composite hydrogel possessed superior moldability. As illustrated in Figure 4f, the PBST-0.01Ag-DFs hydrogel could be readily remolded into diverse shapes, including penguin, whale and dolphin shapes. Such favorable moldability further reflected the remarkable flexibility of the PBST hydrogel, which endowed it with intimate skin conformability and enabled its practical application in epidermal scenarios.

#### 3.1.2. Electrically Driven Drug Release from the Hydrogels

**Figure 5:**
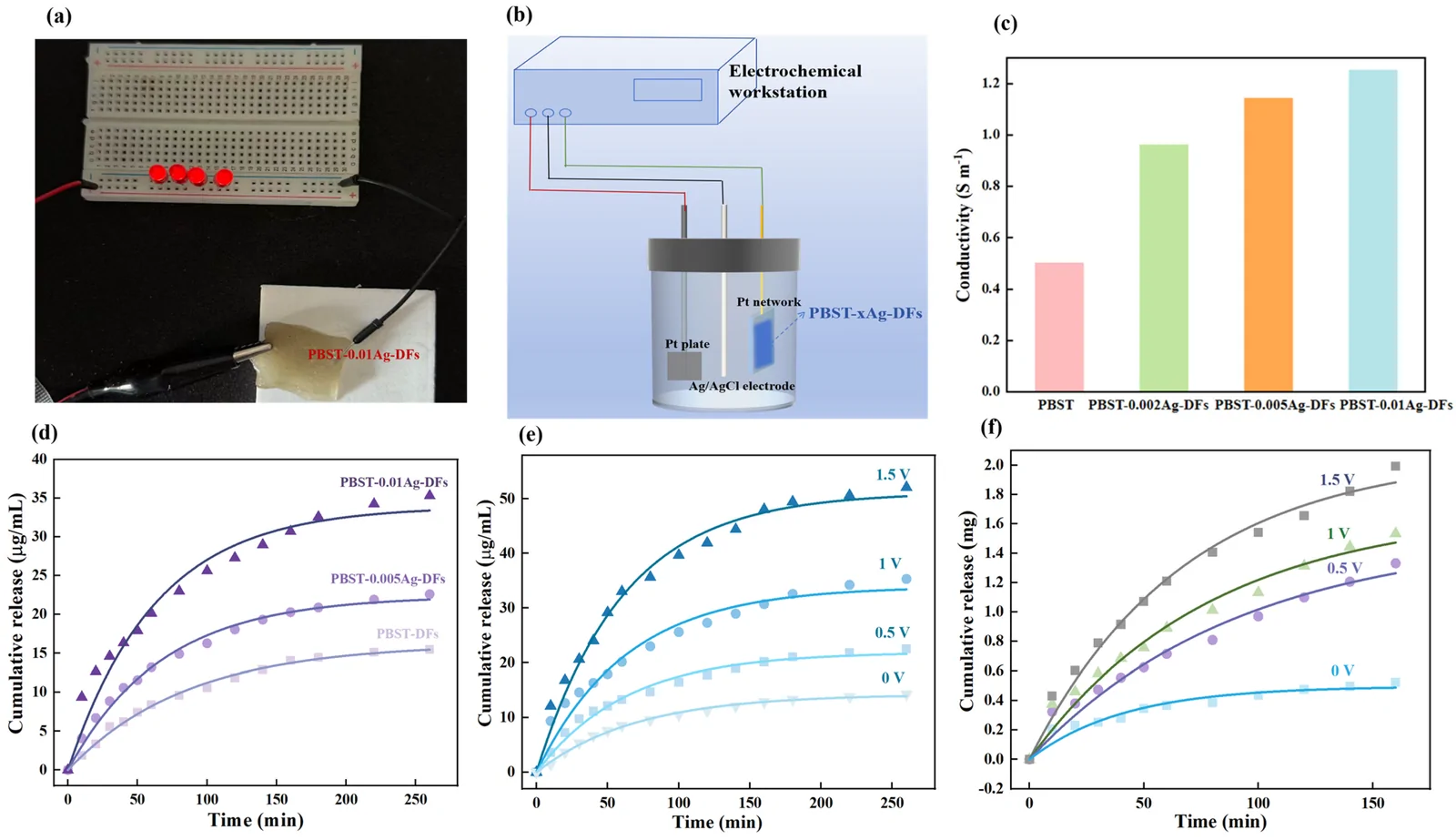
(**a**) Electrical conductivity of PBST-0.01Ag-DFs hydrogel; (**b**) Schematic illustration of electrical conductivity measurement setup; (**c**) Electrical conductivity of hydrogels with different AgNP contents; (**d**) Cumulative release curves of the hydrogels at 1.0 V; (**e**) Accumulated drug release of PBST-0.01Ag-DFs hydrogel at different voltages; (**f**) Release efficiency at different voltages.

Ionic conductivity serves as a critical performance metric for drug-loaded electrodes and constitutes an indispensable prerequisite for the implementation of electro-stimulated drug delivery systems [38,39]. As shown in Figure 5a, as PBST-0.01Ag-DFs hydrogel was connected in the circuit as a conductor, and four light bulbs could be lit, demonstrating its excellent conductivity. Then, the quantitative conductivity of the hydrogels was measured based on the EIS system, illustrated in Figure 5b. The ionic conductivity of PBST-xAg-DFs hydrogels shown in Figure 5c changed in the range of 0.45 to 1.25 S m^−1^, confirming the capability of responding to external electrical stimulation. The ionic conductivity increased with the Ag content, deriving from its excellent conductivity. Compared with conventional hydrogel materials, it exhibits superior electrical conductivity and electrical responsiveness.

Subsequently, the electrochemical workstation was applied to study the controlled drug release behavior of the hydrogel under externally powered electrical stimulation [40]. The hydrogel was adhered on the electrode, and the cumulative release amount of DFs in the buffer was measured. Figure 5d,e show the cumulative drug release profiles under different Ag contents and applied positive voltages. The above release data were further fitted with the Box–Lucas 1 first-order model (Table S1). As shown in Figure 5d, under an applied potential of 1.0 V, the drug release curves of all hydrogel samples exhibited sustained-release characteristics. The release rates of all the samples showed fast to slow change and almost reached equilibrium at about 200 min. Also, the release rate of the samples increased with the content of Ag particles. The hydrogel incorporating 0.01wt% AgNPs exhibited a drug release amount three times that of the non-doped hydrogel. Higher conductivity could also be achieved by altering the voltage. Thus, the cumulative amount and electro-stimulated release efficiency of PBST-0.01Ag-DFs relative to different voltages was determined. The spontaneous release behavior of PBST-DFs without electrical stimulation was insignificant, with a release efficiency of only 10 μg mL^−1^ within 260 min. When a positive potential of 0.5 V was applied, the cumulative drug release was 20.5 μg mL^−1^. As the applied voltage was further increased to 1.5 V, a cumulative release amount of DFs of up to 55 μg mL^−1^ was obtained, about 5 times higher than that only based on drug diffusion. Electrical stimulation dramatically enhanced the drug release rate.

To test the drug release behavior of the hydrogel toward simulated skin, an electrode-attached hydrogel was encapsulated with cellulose membrane, which is widely used as simulated skin tissue. The drug release efficiency of the PBST-0.01Ag-DFs hydrogel across the simulated skin was measured, and the corresponding release curves are shown in Figure 5f. The results align with those obtained from the three-electrode system. The greater the applied electrical stimulation, the higher both the release efficiency and the quantity released. But the release efficiency and quantity in the original time period decreased due to the barrier of the simulated skin. Notably, 0.5 V produced a prominent improvement in the released amount, which may be attributed to the electrical contribution of the cellulose membrane. Kinetic analysis of permeation-based release was also performed using the Box–Lucas 1 first-order model (Table S2).

#### 3.1.3. Release Mechanism of the Hydrogels Based on Electrical Stimulation

**Figure 6:**
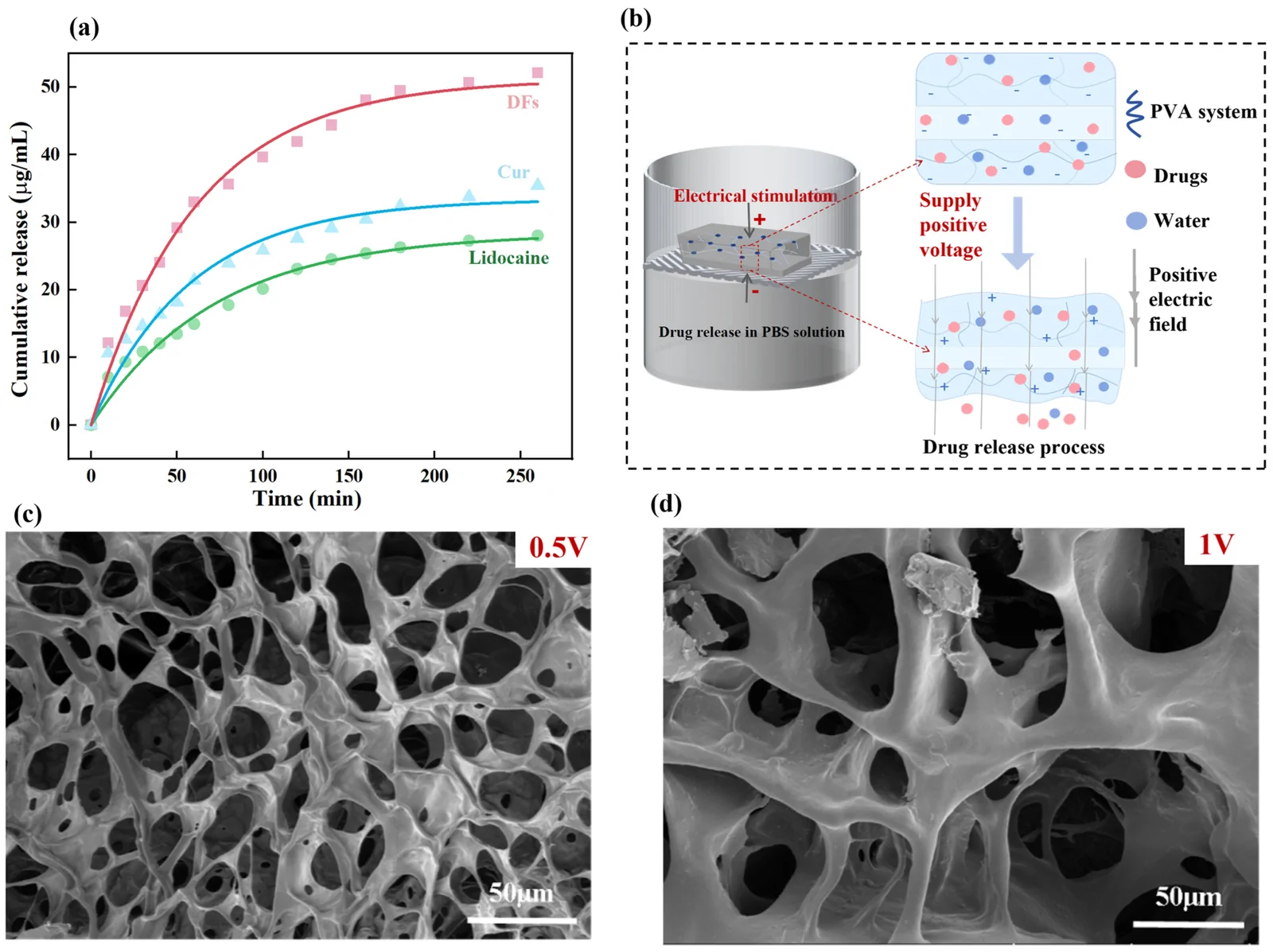
(**a**) Cumulative release profiles of different drugs; (**b**) Schematic diagram of the mechanism of electrically stimulated drug release from conductive hydrogels; (**c**,**d**) SEM morphology of PBST-0.01Ag-DFs hydrogel after treatment at different voltages.

To study the drug release mechanism, the drug release behaviors of three pharmaceuticals with different electrical properties from the hydrogels were investigated. As anticipated, when a positive voltage was applied, positively charged DFs exhibited the highest release rate and quantity, while negatively charged lidocaine hydrochloride showed the lowest, and neutral curcumin presented moderate release performance. All three groups displayed an initial fast-release stage and gradually reached a release plateau, consistent with typical sustained-release characteristics. Box–Lucas 1 first-order kinetic fitting gave adjusted R^2^ > 0.95 for all samples (Table S3), demonstrating that the release profiles statistically follow first-order-like kinetics. Passive diffusion acts as the fundamental release pathway, whereas external electric stimulation disturbs the electrostatic interactions between charged cargoes and hydrogel networks and drives ion migration, further modulating their release in a charge-dependent manner. Figure 6a. Based on the aforementioned research, a mechanism for drug release was proposed, as shown in Figure 6b. As the hydrogel contained abundant hydroxyl and carboxyl groups, numerous negative charges were distributed along its three-dimensional network structure. Thus, strong electrostatic attraction occurred between positively charged DFs drug molecules and the negatively charged network framework, which enabled effective drug loading and immobilization within the hydrogel matrix. Driven by external electrical stimulation, the built-in electric field disrupted the original electrostatic interaction between loaded drugs and hydrogel chains. Accordingly, the dissociation of drugs from the hydrogel was accelerated along with an increased migration rate of drug molecules through the porous network channels, leading to an enhanced drug release amount and release rate compared with the passive diffusion group. In contrast, negatively charged lidocaine hydrochloride was restricted from release due to electrostatic repulsion with the anionic hydrogel network under positive electric field. Without electrostatic interactions with the matrix, neutral curcumin relied merely on passive diffusion and thus achieved intermediate release performance. In addition, morphological observation of the hydrogel after applying different voltages revealed a structural change (Figure 6c,d). The voltage induced significant swelling of pores and fusion of the frame of the network. Thus, the pore size and wall thickness of the hydrogel increased obviously after the application of higher voltage. The structural adjustment might derive from the existence of reversible borate ester bonds. So, the electrically responsive on-demand drug release process of PBST-xAg-DFs primarily relied on the synergistic interaction between electric field-induced network structural changes and charge transmigration. Thus, upon application of a positive voltage, the drugs, which were physically encapsulated within the three-dimensional crosslinked network of the hydrogel, were released gradually from the wall of pores due to the structural adjustment of the hydrogel while undergoing directional migration due to electrically driven stimulation. However, these two mechanisms were tightly coupled within the integrated device, so their individual contributions to total drug release could not be quantitatively separated under existing experimental conditions.

### 3.2. Structure and Piezoelectricity of the Electrospun PVDF

**Figure 7:**
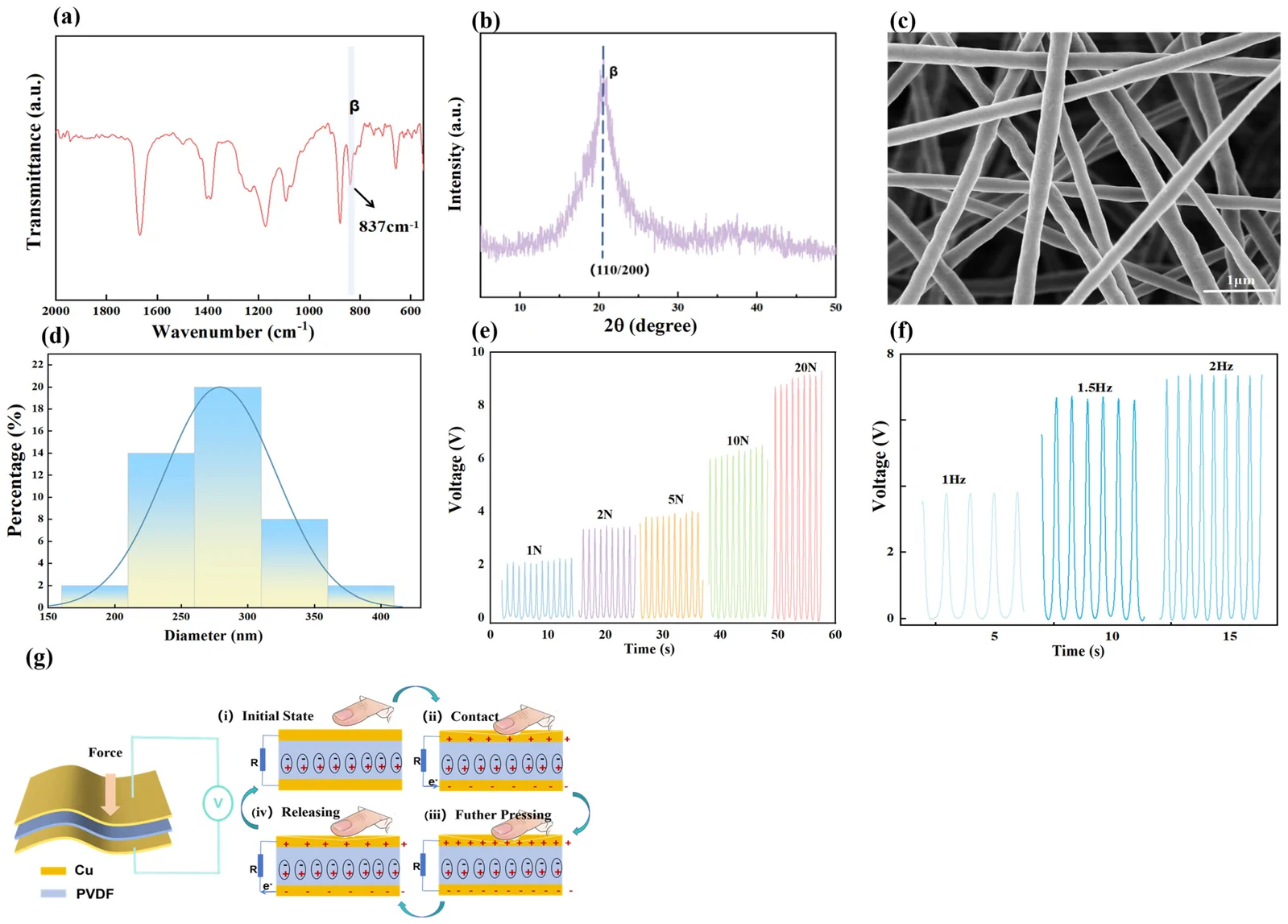
Schematic diagram of electrospun PVDF nanofiber and the structure of PVDF; (**a**) FT-IR spectrum, (**b**) XRD pattern; (**c**) SEM images; (**d**) Diameter; (**e**) The piezoelectric response mechanism of PVDF nanofibers under varying external mechanical forces; (**f**) Different frequencies; (**g**) Schematic diagram illustrating the working principle of PENG.

PVDF is a type of polymorphic synthetic polymer, primarily existing in four crystalline forms: α, β, γ, and δ [41]. In the β phase with an all-trans conformation, it possesses an intrinsic net dipole moment and superior piezoelectric coefficient, rendering it the dominant crystalline phase that governs the output performance of PENG. Electrospinning can regulate molecular polarity reorganization, thereby inducing the growth of the β phase. In the FTIR spectra in Figure 7a, the peak at 837 cm^−1^ was the characteristic signal of β-phase PVDF, assigned to carbon skeleton vibration of CH_2_ and asymmetric stretching of CF_2_, verifying the formation of β phase [42]. According to the FTIR spectra, the β-phase fraction was quantified following the reported Gregorio’s equation [31]. The calculated β-phase content was 47.9%, confirming the high proportion of piezoelectric β crystals within the PVDF matrix. So, β phase apparently existed in the membranes, which was also confirmed by XRD measurement. As shown in Figure 7b, the diffraction peaks at 2θ = 20.3° corresponded to the diffraction of (110) lattice plane of the β phase [43]. The SEM image of the PVDF membrane showed that PVDF nanofiber had a smooth structure and the average diameter of fibers was 160 to 400 nm, Figure 7c,d. The mechanoelectrical response of the aligned PVDF fiber membranes was characterized by assembling them into PENGs. This was achieved by sandwiching the electrospun membrane between two copper electrodes, attaching conducting wires, and encapsulating the entire device with adhesive tape. With application of increasing stress on the PVDF layer from 1 N to 20 N, the Voc gradually increased from 2 Voc to 9 Voc, Figure 7e. While maintaining a pressure of 5 N constantly, the Voc gradually increased from 4 Voc to 8 Voc with the increase in the applied frequency from 1 Hz to 2 Hz, Figure 7f. Compared with previously reported PVDF-based piezoelectric devices, this composite film exhibited superior piezoelectric output performance under conditions of low mechanical force and low-frequency stimulation, offering significant advantages for applications in wearable devices and wound dressings.

The piezoelectric responsiveness of PVDF was due to the existence of dipole moments between carbon and fluorine atoms [44]. When mechanical stress is applied to PVDF, the electric dipole moments within the material arrange themselves in an orderly fashion within the crystalline phase, causing charge separation and accumulation on the material’s surface and thereby generating a voltage, Figure 7g.

### 3.3. Structure and Characterization of Self-Powered PVDF/PBST Wound Dressing

**Figure 8:**
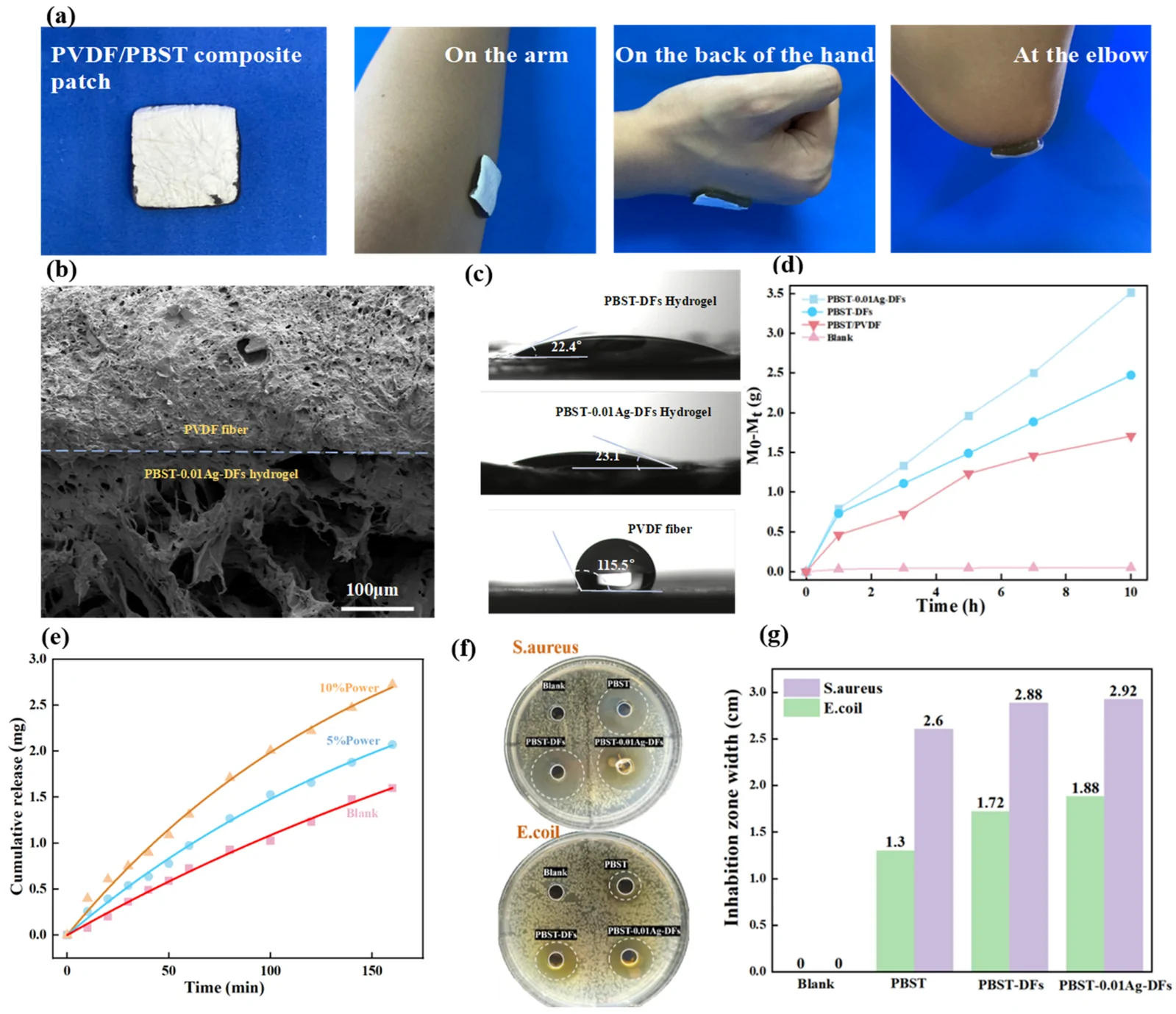
(**a**) Optical photograph of PVDF/PBST wound dressing attached to different areas of human skin; (**b**) SEM image of the sectional structure of the wound dressing; (**c**) Water contact angle of different layers of wound dressing; (**d**) Amount of water adsorbed by dressing; (**e**) Drug release of wound dressing at different pressure frequencies; (**f**,**g**) *E. coli* and *S. aureus* antibacterial zone after co-culture with wound dressings.

In this study, PVDF electrospun layers were integrated with hydrogels to synthesize functional wound dressings. The PVDF layer not only afforded electrical stimulation for the hydrogel based on its piezoelectric property, but also protected the hydrogel from environmental contamination. Moreover, the membrane prevented the dressing’s adhesion to external materials such as clothing. A macroscopic image of the resulting dressing is presented in Figure 8a. The strong adhesion of the wound dressing was demonstrated by sticking it on different parts of human skin, and observing that it did not drop off with movements of the human body. The strong adhesion derived from the interaction of the abundant catechol groups of TA with phenolic hydroxyl groups in SA, as well as the hydroxyl groups of PVA with human skin, in addition to as the stickiness of the hydrogel. The intimate interaction between the PVDF layer and hydrogel layer was examined using SEM (Figure 8b). The image revealed that the PVDF layer exhibited relatively dense packing, while the inner layer featured large pores and loose packing, facilitating the absorption of wound exudate. However, the two layers attached to each other strongly without obvious fracture due to the insertion of hydrogel into the pores of the spun membrane. 

In order to test the adsorption ability as well as the penetrating ability of the dressing toward wound exudate, the contact angles of the dressing’s two sides as well as the adsorbed amount of water were studied, as shown in Figure 8c. The contact angles of the PVDF side and the gel layer of (PBST-0.01Ag-DFs)/PVDF were 115.5° and 23.1°, respectively. The dressing featured asymmetric wettability, with a moderately hydrophobic outer layer blocking contaminants and a fully hydrophilic inner layer absorbing exudate.

Due to the porous structure of both layers, this dressing also exhibited excellent breathability. The breathability test method involved covering a beaker of water with the dressing and measuring its weight change over time. As shown in Figure 8d, the control group (cling film) exhibited virtually no weight change. While PBST hydrogel as well as the Janus (PBST-0.01Ag-DFs)/PVDF all exhibited different degrees of breathability, among which PBST-0.01Ag-DFs showed the highest breathability. The incorporation of silver nanoparticles optimized pore size and connectivity, enhancing gas diffusion. In contrast, PBST/PVDF dressings demonstrated reduced breathability due to the extension of passing routes as well as the increased compactness of the PVDF layer.

To simulate varying pressure conditions in vitro, a gradient of mechanical stress was applied to PVDF/PBST wound dressings using an ultrasonic cell disruptor. As reported in the literature, human body movements apply low-frequency macroscopic bending, stretching and compression to wound dressings [45]. Ultrasound delivers high-frequency non-contact excitation, inducing subtle micro vibration of the piezoelectric film [46,47]. For these reasons, ultrasound was used as an in vitro surrogate to mimic the mechanoelectrical effect induced by human body motion. Ultrasonic vibration can serve to trigger the piezoelectric response of PVDF membranes, converting mechanical vibration into electrical signals. By adjusting the ultrasonic power into medium pressure to simulate body movements, the effect of mechanical force on the drug release performance of hydrogels was studied. As shown in Figure 8e, at different pressure frequencies, the drug release efficiency of PBST/PVDF varies. As pressure increases, the cumulative release amount is accelerated to approximately twice that of natural diffusion. All release curves could be well-fitted by the Box–Lucas 1 kinetic model, and the corresponding fitting parameters are summarized in Table S4. This indicated that ultrasonic piezoelectric stimulation of the PVDF membrane electrically activated the hydrogel, thereby accelerating drug release. Simultaneously, it provided a power supply for wound dressings, laying the foundation for daily applications.

Electrochemical impedance spectroscopy (EIS) was employed to investigate the interfacial electrochemical behavior of conductive hydrogels before and after drug release (Figure S2). The stable solution resistance (Rs) confirmed a steady medium environment without external interference. Drugs embedded in the hydrogel participated in charge conduction, leading to low initial interfacial impedance. After drug release, the loss of conductive drug components and reconstruction of microporous structure hindered charge transfer, increasing the Nyquist semicircle diameter and interfacial charge-transfer resistance (Rct), which verified effective drug release. The low-frequency Warburg impedance indicated a diffusion-controlled release mechanism, further demonstrating that electrical stimulation efficiently regulates interfacial charge transport and mediates drug diffusion and release. As shown in Figure 8f, the zone of inhibition test revealed no antimicrobial activity in the control group. With PBST treatment, the samples exhibited inhibition zones of 2.6 cm and 1.3 cm against *S. aureus* and *E. coli*, respectively, demonstrating the inherent antibacterial activity of TA. Following the incorporation of 0.01wt% AgNPs and DFs, the antimicrobial performance was mostly pronounced. This enhancement stemmed from the synergy of DFs with AgNPs to maximize antimicrobial efficacy.

## 4. Conclusions

This study developed a smart dual-layer wound dressing based on a piezoelectric-conductive synergistic mechanism. The conductive layer was a multifunctional PBST-xAg-DFs hydrogel with Ag particles to enhance the ionic conductivity. When the silver content was 0.01 wt%, the hydrogels’ mechanical properties, self-healing performance and antibacterial efficacy all reached their optimum levels. When an external voltage of 1.5 V was applied, the frame of the network underwent structural adjustment due to the change in dynamic boronated ester. Consequently, drug release was accelerated five-fold under the electricity-driven structural change, thereby achieving on-demand, precise drug delivery. Meanwhile, uniformly dispersed silver nanoparticles exerted broad-spectrum antibacterial effects. On the other hand, the PVDF piezoelectric nanofiber membrane provided a path to convert the mechanical energy of bodily movement to electrical stimulation. The relatively small-diameter fibers and network facilitated a strong connection with the hydrogel and the smooth transfer of electricity. Thus, drug release by the hydrogel was promoted. Through their synergistic action, autonomous electrical stimulation, dynamic repair, real-time antibacterial protection and on-demand drug delivery were realized simultaneously, providing a novel solution for the active repair of hard-to-heal wounds.

## Data Availability

Data are contained within the article.

## Supplementary Materials

The following supporting information can be downloaded at: https://www.mdpi.com/article/10.3390/polym18172175/s1. Figure S1. SEM of the sectional face after self-healing, Figure S2. EIS of the hydrogel-PVDF bilayer dressing before and after drug release. The inset presents the fitted equivalent circuit model, Table S1. Fitting parameters obtained by the Box-Lucas-1 kinetic model for drug-release profiles, Table S2. Fitting parameters obtained by the Box-Lucas-1 kinetic model for drug-release profiles, Table S3. Fitting parameters obtained by the Box-Lucas-1 kinetic model for drug-release profiles, Table S4. Fitting parameters obtained by the Box-Lucas-1 kinetic model for drug-release profiles.
