# Supplementary material for: Self-Driven Drug Release Dual-Layer Dressing Composed of Electrospun Membrane and Hydrogel with Dynamic Structure Based on Piezoelectricity Transformation and Electrical Responsiveness
Source: Polymers (Basel). 2026 Sep 6;18(17):2175. doi: 10.3390/polym18172175 (PMC13568382; doi:10.3390/polym18172175)
Supplement: Supplementary file 1 [file polymers-18-02175-s001.zip › polymers-4474244-supplementary.pdf]

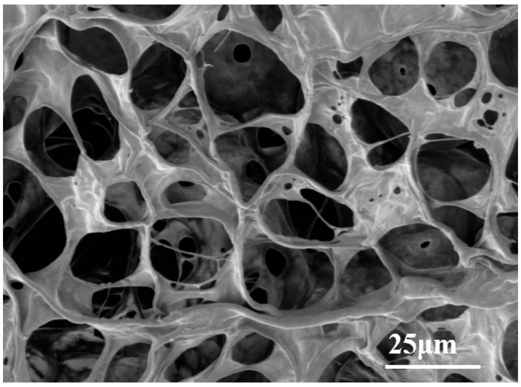

Figure S1. SEM of the sectional face after self-healing.

Table S1. Fitting parameters obtained by the Box-Lucas-1 kinetic model for drug-release profiles.

sample	a	b	R^2
PBST-DFs	22.3814	0.0148	0.9913
PBST-0.005Ag-DFs	33.947	0.0159	0.963
PBST-0.01Ag-DFs	22.3814	0.0148	0.9913
0 V	14.2843	0.0145	0.9952
0.5 V	21.9397	0.016	0.9846
1.0 V	33.947	0.0159	0.963
1.5 V	51.199	0.0164	0.986

* Drug release profiles were fitted according to the equation $y = a(1-e^{-bx})$. Drug release with different Ag and under different applied voltages (μg mL⁻¹)

Table S2. Fitting parameters obtained by the Box-Lucas-1 kinetic model for drug-release profiles.

sample	a	b	R^2
0 V	0.4975	0.0244	0.9287
0.5 V	1.5284	0.011	0.9675
1.0 V	1.6945	0.0127	0.9731
1.5 V	2.0756	0.0148	0.9858

* Permeation-based drug release under different applied voltages (mg)

Table S3. Fitting parameters obtained by the Box-Lucas-1 kinetic model for drug-release profiles.

sample	a	b	R^2
DFs	51.2	0.016	0.986
Cur	33.44	0.016	0.9574
Lidocaine	28.4	0.014	0.9746

* Cumulative release of different drugs was determined in buffer solution (μg mL^{-1}).

Table S4. Fitting parameters obtained by the Box-Lucas-1 kinetic model for drug-release profiles.

sample	a	b	R^2
10% Power	4.2173	0.0064	0.9951
5% Power	3.6959	0.0051	0.9968
Blank	4.2699	0.0029	0.9958

* Drug Release of wound dressing at Different Pressure Frequencies (mg).

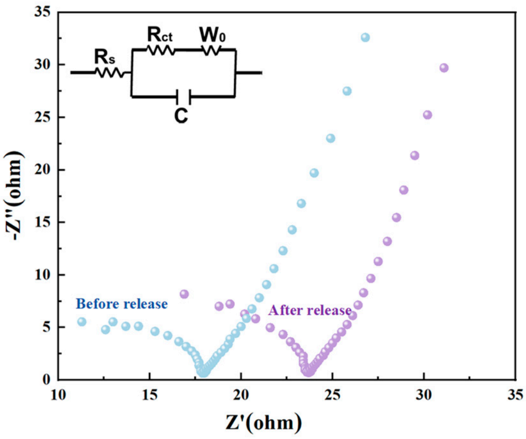

Figure S2. EIS of the hydrogel-PVDF bilayer dressing before and after drug release. The inset presents the fitted equivalent circuit model.
